# Contemporary non-statin therapies for dyslipidemia management: achieving current lipid targets

**DOI:** 10.3389/fmed.2026.1894845

**Published:** 2026-08-24

**Authors:** Carlos I. Ponte-Negretti, Alberto Lorenzatti, Fernando Wyss-Quintana, Rodrigo Alonso, Máxima Méndez-Castillo, Osiris Valdez-Tiburcio, Vladimir E. Ullauri-Solórzano, Camila Y. Ullauri-Valcárcel, Adriana Puente-Barragán, Luz C. Zárate-Correa, José G. Prieto-Marín, Isaac A. Suárez Sangucho, Jorge Vasconez-Gonzalez, Esteban Ortiz-Prado

**Affiliations:** 1 Unidad De Medicina Cardiometabólica, Instituto Médico La Floresta, Caracas, Venezuela; 2 Servicio De Cardiología, Fundación Rusculleda De Investigación En Medicina, Instituto Médico DAMIC, Códoba, Argentina; 3 Unidad De Cardiología Cardio Solutions, Guatemala City, Guatemala; 4 Unidad Cardiovascular, Centro Avanzado De Medicina Metabólica y Nutrición, Santiago, Chile; 5 Unidad De Lípidos, Clínica De Lípidos CLL LIPID, Santo Domingo, Dominican Republic; 6 Departamento De Cardiología, Hospital Central Romana, La Romana, Dominican Republic; 7 Departamento De Cardiología, Hospital Metropolitano, Quito, Ecuador; 8 Unitat De Recerca, Clínic BarceLona-Institut d’Investigacions Biomèdiques August Pi Sunyer (IDIBAPS), Barce-Lona, Spain; 9 Departamento De Cardiología, Hospital Centro Médico Nacional 20 De Noviembre, Instituto De Seguridad y Ser-Vicios Sociales De Los Trabajadores Del Estado, Mexico City, Mexico; 10 Unidad De Cardiología, CardioDec, Cali, Colombia; 11 One Health Research Group, Universidad De Las Americas, Quito, Ecuador

**Keywords:** cardiovascular prevention, dyslipidemia, LDL-C, non-statin therapies, precision medicine, risk stratification, statin intolerance

## Abstract

The current treatment of dyslipidemia in patients with cardiovascular risk (CR) is based on three well-defined principles: First, given the extensive evidence demonstrating the association between elevated low-density lipoprotein cholesterol (LDL-C) levels and cardiovascular risk, the primary therapeutic target is LDL-C. Second, as current guidelines recommend LDL-C targets below 55 mg/dL for high-risk patients, therapeutic goals according to risk level should be achieved as early as possible, and combination therapy is increasingly necessary. Third evidence indicates that non-statin therapies—including ezetimibe, bempedoic acid, PCSK9 inhibitors (evolocumab and alirocumab), and inclisiran—provide substantial LDL-C reductions and reduce cardiovascular events. These agents are particularly effective in very high-risk and statin-intolerant populations, with bempedoic acid addressing both lipid and inflammatory pathways. Additionally, the fixed-dose combination of bempedoic acid and ezetimibe produces an approximately 36.2% reduction in LDL-C in high-risk patients. Risk-stratified guidelines recommend ezetimibe as first-line add-on therapy, followed by PCSK9 inhibitors or bempedoic acid based on residual LDL-C levels and tolerability. Alirocumab has demonstrated a significant reduction in the primary composite endpoint of major adverse cardiovascular events (MACE), with a favorable trend in all-cause mortality. Furthermore, inflammation (measured by hsCRP) provides complementary prognostic information beyond LDL-C. This expert position paper proposes practical algorithms for a precision medicine approach in Latin America, matching treatment intensity to individual risk profiles for the primary and secondary prevention of atherosclerotic cardiovascular disease.

## 1 Introduction

Dyslipidemia is traditionally defined by specific laboratory cutoffs, including LDL-C ≥ 130 mg/dL, triglycerides ≥ 150 mg/dL, or HDL-C < 40 mg/dL (1). However, contemporary guidelines shift the focus from static diagnostic thresholds to risk-based management, wherein treatment goals for atherogenic lipoproteins are stratified according to individual absolute cardiovascular risk (2). Elevated low-density lipoprotein cholesterol (LDL-C) levels are associated with increased cardiovascular risk (3), hence even small reductions in LDL-C levels sustained over long periods of time translate into a reduction in cardiovascular risk (4). The epidemiology of dyslipidemia varies by region, age, sex, and ethnicity, and is influenced by genetic and environmental factors. According to data from a systematic review that analyzed 206 studies, the estimated global prevalence was 28.8% for hypertriglyceridemia, 24.1% for hypercholesterolemia, 38.4% for low HDL-C, and 18.93% for high LDL-C (5). It has been observed that men show a higher prevalence of hypertriglyceridemia, while women tend to have higher HDL cholesterol levels and lower LDL cholesterol levels (5, 6).

The pathophysiology of atherosclerosis involves multiple factors, among which elevated LDL-C concentration is one of the most important (7). Current guidelines recommend increasingly strict LDL-C targets according to cardiovascular risk categories, often necessitating the use of combination therapies with complementary mechanisms of action (8, 9). The IMPROVE-IT trial in 2015 demonstrated that the addition of the non-statin agent ezetimibe reduces major adverse cardiovascular events, resulting in a reduction of baseline LDL-C from 2.4 mmol/L to 1.4 mmol/L (10). Residual cardiometabolic risk reduction should employ therapeutic options adapted to specific patient needs, considering factors such as statin intolerance, inflammatory risk, and socioeconomic determinants, through personalized medicine and patient-centered decision-making (11). Furthermore, the ALALIP Consensus establishes five therapeutic targets for cardiovascular risk reduction: triglyceride-rich lipoproteins, inflammation, impaired glucose metabolism, hypertension, and a prothrombotic state (11). This expert position paper evaluates the role of ezetimibe, bempedoic acid, PCSK9 inhibitors, and inclisiran in dyslipidemia management, proposing practical algorithms for primary/secondary prevention and statin intolerance.

## 2 Scope of the positioning

This document is an expert positioning, not a comprehensive dyslipidemia manual, clinical practice guideline, or consensus. Recommendations were developed without formal grading but achieved > 80% acceptance among coauthors developing the modified Delphi methodology. The focus is on LDL-C as the primary target; management of triglycerides, non-HDL-C, and lipoprotein(a) is beyond scope. Pharmacoeconomic analyses are not included, as costs vary widely in Latin America; recommendations must adapt to local resources, epidemiology, and regulations. Emphasis is placed on individualized risk reduction through shared decision-making, considering accessibility and patient preferences.

## 3 Methods

A panel of Latin American experts in lipidology and cardiovascular prevention was convened by ALALIP, SCCH, IAS, ISCP, and CPE. Experts conducted a structured literature review. For this purpose, the databases PubMed, SciELO, Scopus, Web of Science, and LILACS were used. Inclusion criteria: adult patients with dyslipidemia/ASCVD; interventions (ezetimibe, bempedoic acid, PCSK9 inhibitors, inclisiran); outcomes (LDL-C reduction, MACE, mortality, statin intolerance); precision medicine focus; ≥ 12 weeks follow-up.

The scientific evidence identified in the literature that met the inclusion criteria served as the foundation for the development of the Delphi statements. Initially, the experts were organized into pairs to review the selected evidence and prepare preliminary proposals. These proposals were subsequently evaluated through three rounds of consensus involving the full expert panel. Recommendations were accepted when they achieved an agreement level of greater than 80% among panel members. For proposals that did not reach the predefined consensus threshold, the panel’s observations and comments were incorporated to revise the statements. Revised proposals were reassessed in subsequent Delphi rounds, and those that still failed to achieve at least 80% agreement were permanently discarded.

## 4 Mechanisms of action

Non-statin therapies act on multiple targets across cholesterol metabolism to improve LDL-C levels. The following table summarizes their mechanisms of actions, the magnitude of LDL-C reduction, and their indications within a precision medicine approach (Table 1). Precision medicine refers to the tailoring of treatments to subgroups of patients who share a common susceptibility to a specific disease or exhibit a similar response to a particular drug (12). This therapeutic approach is not intended to replace recommendations from international clinical guidelines; rather, it aims to complement them through a comprehensive patient assessment to determine when early treatment intensification is warranted, when combination therapy should be implemented, and which patients are most likely to benefit from one therapeutic agent over another.

**TABLE 1:** Pharmacologic lipid-lowering therapies and their mechanisms, efficacy, and precision medicine indications.

Therapy	Mechanism of action	LDL-C Reduction (%)	Precision medicine indications
Ezetimibe	Inhibits intestinal cholesterol absorption via NPC1L1	20–25 (17, 18)	First-line add-on; hyperabsorbers; cost-effective in Latin America (10, 11, 43)
Bempedoic Acid	Inhibits ATP-citrate lyase in liver, reducing cholesterol synthesis	Monotherapy: 17–28 (18, 19) Combination with ezetimibe: 36,2 (fixed-dose combination) (18, 19)	Statin intolerance; dual anti-inflammatory effect (35, 58)
Evolocumab (PCSK9 inhibitor)	Monoclonal antibody blocking PCSK9, increasing hepatic LDL receptors	59–61.09 (9, 24)	Very high-risk; genetic mutations in PCSK9/LDLR (24, 59)
Alirocumab (PCSK9 inhibitor)	Monoclonal antibody blocking PCSK9	46–62 (9, 24)	Post-ACS; mortality benefit (all-cause mortality reduction) (27)
Inclisiran	siRNA silencing PCSK9 gene in liver	49.9–52.3 (15)	Adherence issues; twice-yearly dosing (28).

**FIGURE 1:**
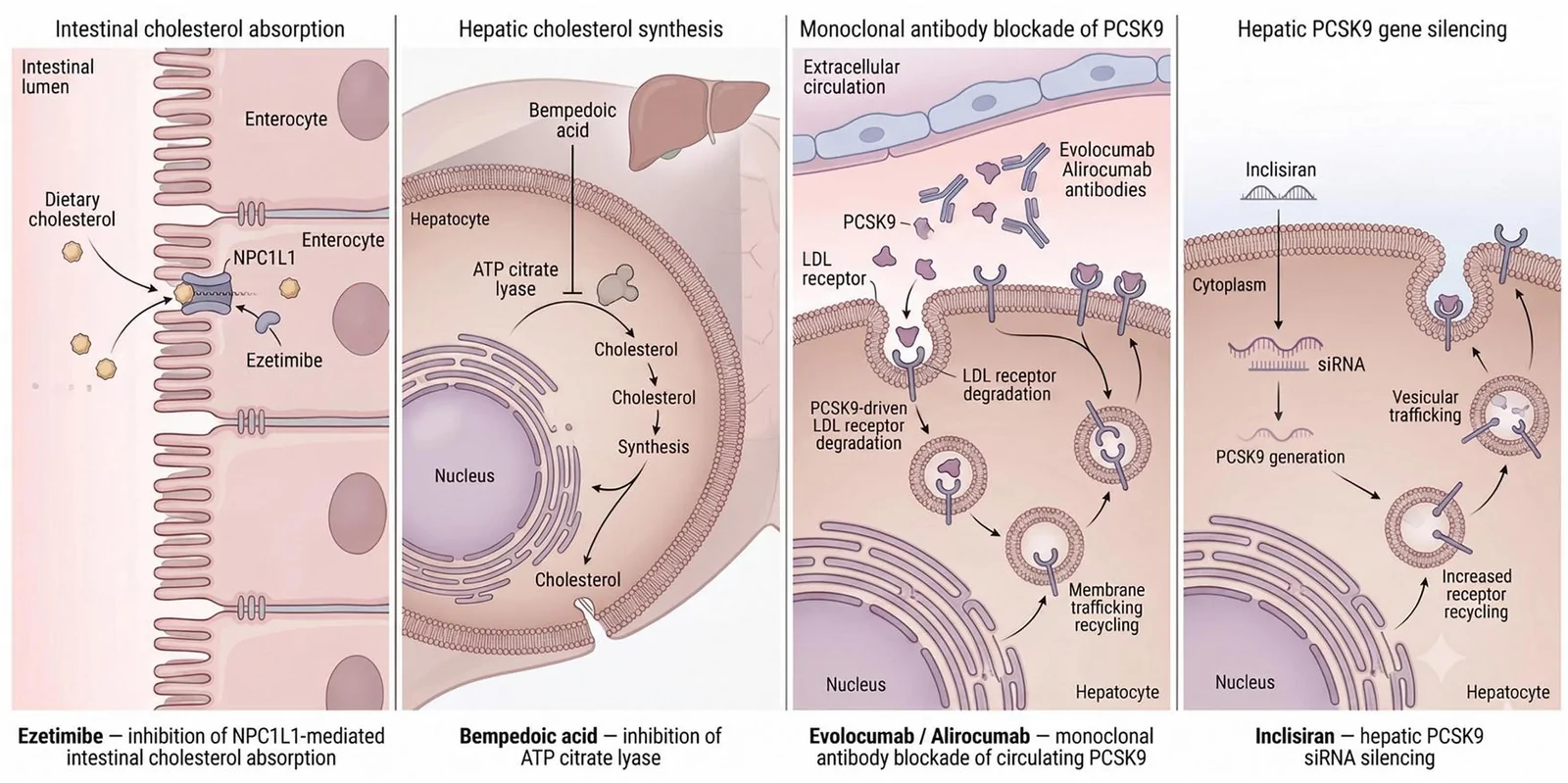
Mechanisms of action of non-statin therapies across cholesterol metabolism. An integrated diagram illustrating four therapeutic strategies to lower lipid levels: (1) Ezetimibe inhibits intestinal cholesterol absorption by blocking the NPC1L1 transporter on enterocytes. (2) Bempedoic acid reduces hepatic cholesterol synthesis by inhibiting the upstream enzyme ATP citrate lyase (ACL). (3) Evolocumab and Alirocumab are monoclonal antibodies that bind circulating extracellular PCSK9, preventing it from targeting LDL receptors for degradation and enhancing receptor recycling. (4) Inclisiran uses small interfering RNA (siRNA) to silence hepatic PCSK9 generation intracellularly, leading to reduced PCSK9 production and increased LDL receptor recycling.

Building on these data, Figure 1 provides an integrated visual summary of the main non-statin therapies used in contemporary dyslipidemia management. It illustrates their distinct mechanisms of action across cholesterol metabolism, the approximate magnitude of LDL-C reduction achieved by each agent, and their place within a risk-guided precision medicine treatment framework (Figure 1).

## 5 Clinical evidence

Non-statin lipid-lowering therapies can be used in different clinical scenarios to achieve LDL-C goals (Table 2).

**TABLE 2:** Therapeutic strategies for LDL-C reduction according to clinical scenario and cardiovascular risk level.

Clinical scenario	Preferred therapy	Rationale	Expected LDL-C reduction (%)	Key evidence	Precision medicine consideration
Not at goal on maximally tolerated statin	Ezetimibe (first add-on)	Oral, safe, cost-effective, guideline-recommended first step	20–23%	IMPROVE-IT	First-line add-on in most patients; widely accessible
Very high-risk ASCVD persistent LDL-C ≥ 55 mg/dL	PCSK9 inhibitor (evolocumab / alirocumab)	Largest LDL-C reduction; strong outcome data	50–60%	FOURIER, ODYSSEY OUTCOMES	Preferred when large LDL-C reduction required
Post-acute coronary syndrome (post-ACS)	PCSK9 inhibitor (early addition)	Reduces MACE and mortality in high-risk setting	50–60%	ODYSSEY OUTCOMES	Consider early escalation for rapid risk reduction
Statin intolerance (confirmed)	Bempedoic acid ± Ezetimibe	Oral alternative; effective without muscle-related side effects	17–28% (mono); 36,2% (combo)	CLEAR Outcomes, CLEAR Serenity	Ideal in patients with muscle symptoms or statin intolerance
Persistent LDL-C elevation despite statin + ezetimibe	PCSK9 inhibitor or Inclisiran	Additional potent LDL-C lowering	∼50–60%	FOURIER, ORION trials	Choice guided by adherence, access, and preference
Poor adherence to daily therapy	Inclisiran	Twice-yearly dosing improves adherence	∼50%	ORION-9, 10, 11	Ideal for patients with low adherence or complex regimens
Elevated hsCRP / residual inflammatory risk	Bempedoic acid	Reduces both LDL-C and inflammation	∼20–30% LDL-C; ↓hsCRP ∼20–30%	CLEAR Outcomes	Consider when inflammation contributes to residual risk
Familial hypercholesterolemia / very high baseline LDL-C	PCSK9 inhibitor	Strong LDL-C reduction beyond statins	50–60%	Multiple RCTs	Consider genetic profile and high baseline LDL-C
Limited access / cost constraints (Latin America)	Ezetimibe ± Bempedoic acid	More accessible oral options	20–40%	Multiple trials	Cost and availability are key determinants
Preference against injections	Ezetimibe or Bempedoic acid	Oral administration improves acceptability	20–40%	Multiple trials	Route of administration influences adherence

## 6 LDL-C reduction magnitude

It is important to emphasize that, among the currently available therapies for LDL-C management, PCSK9 inhibitors provide the greatest reductions, approximately 63.0% with evolocumab and 62% with alirocumab, highlighting their importance in lipid-lowering strategies (13, 14). On the other hand, in the ORION-10 trial, inclisiran reduced LDL-C levels by 52.3%, while a 49.9% reduction was observed in the ORION-11 trial (15). In special populations, such as patients with diabetes mellitus, PCSK9 inhibitors have been shown to reduce LDL-C by 48.20%, non–HDL-C by 45.23%, total cholesterol by 30.39%, triglycerides by 11.96%, lipoprotein(a) by 27.87%, and apolipoprotein B by 42.43% (16). Although ezetimibe alone yields consistent yet modest LDL-C reductions of approximately 20–23% (17, 18), dual therapy can provide substantially greater lowering. Bempedoic acid illustrates this principle: it reduces LDL-C by up to 28% as monotherapy and by approximately 36% when combined with ezetimibe-, thereby improving overall dyslipidemia control (18, 19). Finally, a meta-analysis demonstrated that both evolocumab and alirocumab are not only more effective in lowering LDL-C levels, but also reduce the risk of major adverse cardiovascular events (MACE) and atherosclerotic cardiovascular disease (ASCVD), thereby improving patient outcomes (20, 21).

On the other hand, recent evidence has emphasized the importance of combination lipid-lowering therapy in selected patients at high and very high cardiovascular risk, with increasing support for the early initiation of combination therapy, particularly the combination of a high-intensity statin with ezetimibe. The 2024 recommendations from the International Lipid Expert Panel on lipid-lowering therapy in patients with established atherosclerotic cardiovascular disease and following an acute coronary syndrome recommend that combination therapy should be considered from the outset in all patients at very high and extremely high cardiovascular risk (20).

In a phase IV study, the lipid-lowering effect of combination therapy was significantly greater than that of statin monotherapy. Combination therapy enabled a significantly higher proportion of patients to achieve LDL-C targets of < 55 mg/dL (55.0% vs. 15.4%; *P* < 0.0001) and < 70 mg/dL (85.0% vs. 58.5%; *P* = 0.0009) compared with monotherapy (22). Furthermore, initial combination therapy was associated with a significant reduction in all-cause mortality compared with statin monotherapy (OR 0.526, 95% CI 0.378–0.733) and resulted in an absolute risk reduction of 4.7% after 3 years of follow-up (23).

## 7 Cardiovascular outcomes

Regarding the reduction in the risk of major adverse cardiovascular events (MACE), both PCSK9 inhibitors and ezetimibe have been shown to significantly reduce this risk. In a systematic review and meta-analysis evaluating both PCSK9 inhibitors, evolocumab and alirocumab, evolocumab was associated with a significant reduction in the risk of myocardial infarction (MI) (OR 0.72, 95% CI 0.64–0.81; *p* < 0.01), coronary revascularization (OR 0.77, 95% CI 0.70–0.84; *p* < 0.01), stroke (OR 0.79, 95% CI 0.66–0.94; *p* = 0.01), and major adverse cardiovascular events (MACE) (OR 0.85, 95% CI 0.80–0.89; *p* < 0.01). Similarly, alirocumab significantly reduced the risk of MI (OR 0.57, 95% CI 0.38–0.86; *p* = 0.01), cardiovascular mortality (OR 0.35, 95% CI 0.16–0.77; *p* = 0.01), all-cause mortality (OR 0.60, 95% CI 0.43–0.84; *p* < 0.01), and overall MACE (OR 0.35, 95% CI 0.16–0.77; *p* = 0.01) (24). In the FOURIER clinical trial, the results demonstrated that evolocumab significantly reduced the risk of cardiovascular death, myocardial infarction, stroke, hospitalization for unstable angina, and coronary revascularization (21). On the other hand, evolocumab has been shown to reduce the rate of cardiovascular events in high-risk patients with diabetes, regardless of the presence or absence of atherosclerotic cardiovascular disease (25).

In the ODYSSEY OUTCOMES clinical trial, treatment with alirocumab was associated with lower risks of any coronary heart disease event, major coronary events, death from coronary heart disease, nonfatal myocardial infarction, and nonfatal ischemic stroke (26). On the other hand, bempedoic acid reduces cardiovascular risk by 13% when a 21% reduction in LDL-C is achieved (18). In the CLEAR Outcomes randomized trial, patients with diabetes experienced significant reductions in both relative and absolute cardiovascular risk for the four-component major adverse cardiovascular events (MACE-4) endpoint (HR 0.83; 95% CI 0.72–0.95), corresponding to an absolute risk reduction of 2.4% (27). Similarly, exploratory analyses from pooled ORION trials suggest a potential reduction in cardiovascular risk with inclisiran, although dedicated outcomes trials are ongoing (28).

## 8 Evidence in statin-intolerant populations

It is important to consider that in patients unable to tolerate at least two different statins (one at the lowest dose), statin intolerance should be suspected and alternative therapeutic strategies should be considered (9). Statin intolerance represents a significant clinical challenge that limits the effective treatment of patients at risk for or with established cardiovascular disease. According to a comprehensive meta-analysis of 176 studies encompassing 4,143,517 patients, the overall pooled prevalence of statin intolerance is 9.1% (95% confidence interval 8.0–10%) (29). Consistent with the 2026 ACC/AHA Guideline on the management of dyslipidemia, statin intolerance is defined as the inability to tolerate at least two different statins with one statin attempted at the lowest starting daily dose, symptoms typically muscle-related leading to unwillingness to continue therapy, symptoms that resolve upon statin discontinuation and recur with rechallenge, and the absence of other identifiable causes such as vitamin D deficiency, hypothyroidism, or drug interactions (30).

In statin-intolerant patients requiring additional lipid lowering, the combination of bempedoic acid 180 mg daily plus ezetimibe 10 mg daily provides robust LDL-C reduction. In a phase 3 trial by Ballantyne et al., this fixed-dose combination administered for 12 weeks to high-risk patients on maximally tolerated statin therapy reduced LDL-C by 36.2% compared to a 1.8% increase with placebo (placebo-corrected difference −38.0%, *P* < 0.001), and was significantly more effective than ezetimibe alone (−23.2%, *P* < 0.001) or bempedoic acid alone (−17.2%, *P* < 0.001) (31). Importantly, in the CLEAR Serenity trial specifically enrolling 345 patients with statin intolerance, bempedoic acid monotherapy reduced LDL-C by 21.4% compared to placebo at 12 weeks (placebo-corrected difference, 95% CI −25.1% to −17.7%; *P* < 0.001) with numerically fewer muscle-related adverse events in the bempedoic acid group (12.8% vs. 16.2%) (32). Response should be assessed after 4 to 12 weeks, and if LDL-C goal is achieved, the current regimen should be continued with periodic monitoring. For patients not achieving goal on bempedoic acid plus ezetimibe, addition of a PCSK9 inhibitor should be strongly considered. In statin-intolerant populations, PCSK9 inhibitors achieve LDL-C reductions of 53% to 56% with minimal muscle symptoms, as confirmed in the GAUSS-2 trial (33). On the other hand, in patients with statin intolerance, PCSK9 inhibitors (evolocumab and alirocumab) have demonstrated substantial LDL-C reductions of approximately 50–60%, which are associated with a decreased risk of cardiovascular events (9). Finally, a study evaluating the efficacy of inclisiran demonstrated that this medication is equally effective across various backgrounds (34).

**FIGURE 2:**
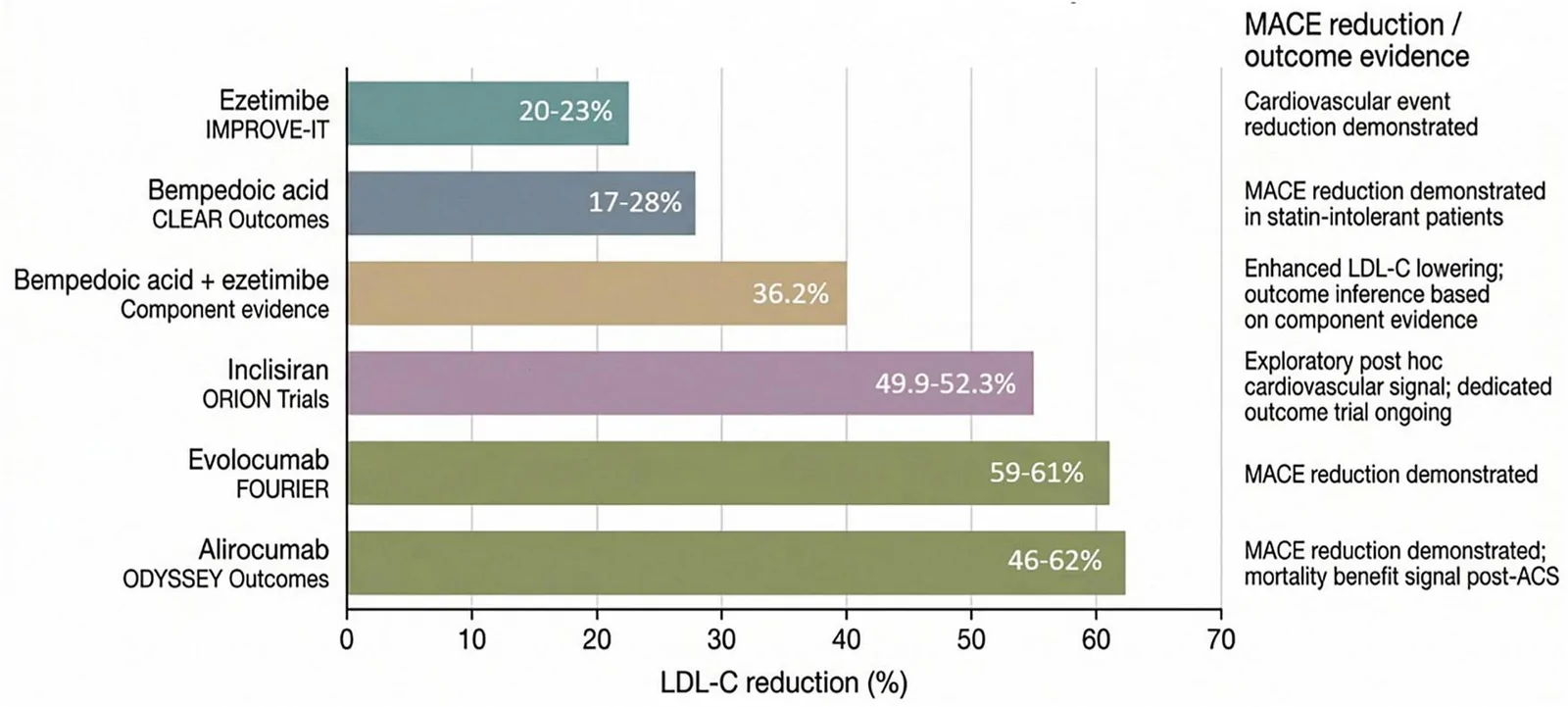
LDL-C reduction and evidence of cardiovascular risk reduction with different lipid-lowering therapies. Comparison of the magnitude of LDL-C reduction achieved with ezetimibe, bempedoic acid, the combination of bempedoic acid and ezetimibe, inclisiran, evolocumab, and alirocumab, together with the available evidence for reduction in MACE.

To translate these principles into clinical practice, Figure 2 summarizes the approximate LDL-C–lowering efficacy of major non-statin therapies and integrates their use into a risk-guided precision treatment algorithm.

## 9 Residual inflammatory risk

Vascular inflammation has been identified as a key determinant of atherosclerotic risk in patients with statin intolerance. Furthermore, it is unlikely that therapies targeting low-density lipoprotein cholesterol (LDL-C) alone are sufficient to completely mitigate the risk of atherosclerosis (35). Studies have shown that although LDL-C levels are strong predictors of cardiovascular events, high-sensitivity C-reactive protein (hsCRP) is also an important predictor among patients with statin intolerance. In this context, bempedoic acid has demonstrated reductions of 21.6% in hsCRP and 21.1% in LDL-C (35). Similarly, Bempedoic acid has been shown to reduce LDL-C by 21% and hsCRP by 22%, resulting in a 13% reduction in cardiovascular risk, further supporting its role in cardiovascular risk reduction (36, 37). In a clinical trial evaluating canakinumab, anti-inflammatory therapy was associated with a significantly lower rate of recurrent cardiovascular events. Similarly, clinical trials have shown that colchicine therapy is associated with a reduced risk of cardiovascular events (38–40). These findings support the use of anti-inflammatory therapy as a strategy to reduce residual cardiovascular risk.

## 10 Therapeutic algorithms

Contemporary management of dyslipidemia is based on three core principles that underpin the therapeutic algorithms presented in this section: First, low-density lipoprotein cholesterol (LDL-C) remains the primary therapeutic target. Second, risk-stratified LDL-C goals should be achieved as early as possible to minimize cumulative exposure and lifetime cardiovascular risk. Third, combination therapy is increasingly required, particularly in individuals with high and very high cardiovascular risk. The algorithms proposed herein translate these principles into pragmatic, stepwise decision pathways that align treatment intensity with individual cardiovascular risk profiles. They incorporate the expanding spectrum of non-statin therapies within a precision medicine framework, enabling a more individualized and effective approach to lipid management.

**FIGURE 3:**
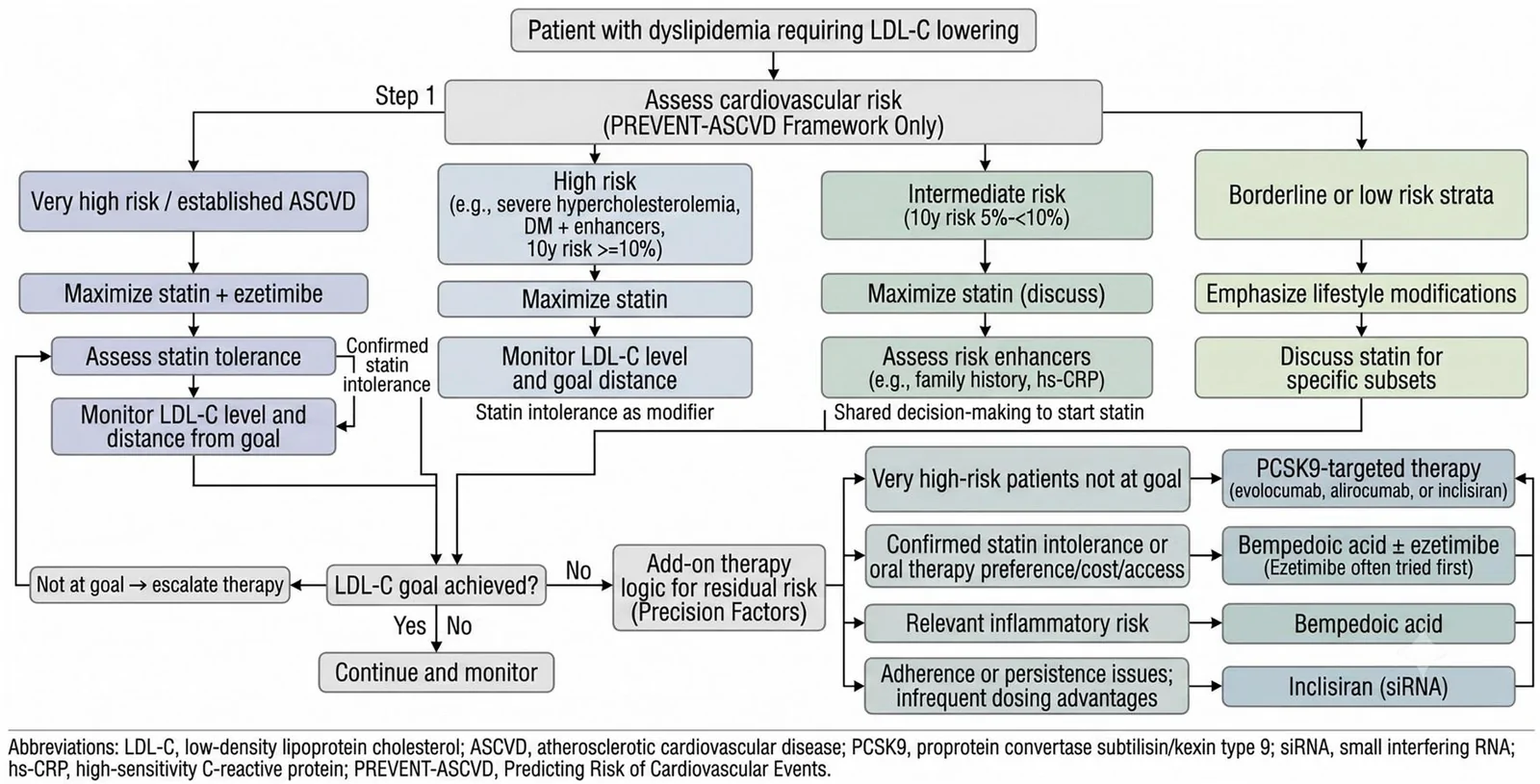
Risk-guided precision algorithm for non-statin therapy selection in dyslipidemia management. The algorithm illustrates a stepwise approach to the selection and escalation of non-statin therapies according to cardiovascular risk within the PREVENT-ASCVD framework. After initial risk stratification, treatment decisions are guided by LDL-C level and distance from goal, statin tolerance, residual inflammatory risk, adherence and persistence, route-of-administration preference, and treatment accessibility.

The following algorithms provide a practical framework for dyslipidemia management, integrating cardiovascular risk stratification, LDL-C thresholds, statin tolerance, inflammatory burden, adherence and persistence, and treatment accessibility. Figure 3 presents a risk-guided precision algorithm for non-statin therapy selection in dyslipidemia management.

## 11 Dynamic risk assessment

The entry point for all therapeutic algorithms in dyslipidemia management relies on a precise and up-to-date estimation of cardiovascular risk. Cardiovascular disease risk assessment in apparently healthy individuals, as well as in older adults, patients with established ASCVD, and those with diabetes mellitus, can provide valuable information to guide individualized interventions (41). Identifying patients at higher risk is a key step in preventing recurrent events. It enables the implementation of more intensive treatments and closer follow-up strategies, promoting more personalized care and improving health outcomes, with significant benefits for both patients and public health (41).

In this context, the 2026 ACC/AHA/AACVPR/ABC/ACPM/ ADA/AGS/APhA/ASPC/NLA/PCNA Guideline on the Management of Dyslipidemia recommends the PREVENT (Predicting Risk of cardiovascular disease EVENTs) equations as the preferred tool, in adults aged 30 to 79 years without ASCVD or subclinical atherosclerosis, replacing the older Pooled Cohort Equations (31). This model introduces a more comprehensive approach by incorporating traditional risk factors such as renal function, metabolic status, and social determinants of health, allowing a more individualized and precisely risk estimation. PREVENT also captures non-atherosclerotic events, including heart failure, thereby better reflecting the overall cardiovascular burden (30).

Risk stratification is defined according to 10-year estimates, with categories of low ( < 3%), borderline (3% to <5%), intermediate (5% to <10%), and high ( ≥ 10%) risk, while very high-risk status requires specific criteria beyond the 10-year risk estimate and is determined by clinical history of multiple major ASCVD events ( ≥ 2), such as recent acute coronary syndrome (within the past 12 months), prior myocardial infarction, ischemic stroke, symptomatic peripheral artery disease, or the coexistence of one major ASCVD event and additional multiple high-risk conditions including advanced age (65 years or older), diabetes, hypertension, heart failure, or persistently elevated LDL-C ( ≥ 100 mg/dL) despite optimized lipid-lowering therapy (30).

Risk-enhancing factors should be actively incorporated into a dynamic clinical evaluation, among individuals classified as borderline or intermediate risk, where they may meaningfully modify overall risk and influence therapeutic decisions. These include a family history of premature ASCVD, elevated lipoprotein(a), persistently increased triglyceride levels, metabolic syndrome, chronic kidney disease, and chronic inflammatory disorders such as psoriasis, rheumatoid arthritis, or HIV infection. Sex-specific conditions, including premature menopause and pregnancy-related complications such as preeclampsia, as well as high-risk ethnic backgrounds. Furthermore, selected biomarkers such as elevated high-sensitivity C-reactive protein, apolipoprotein B, and an ankle-brachial index below 0.9, may provide additional refinement of risk stratification. The presence of these factors reflects an increased cardiovascular risk, strengthens individualized risk profiling, and supports a more proactive and intensive lipid-lowering approach (30).

In selected patients with borderline or intermediate estimated risk with no prior ASCVD, particularly when decision regarding LLT remains uncertain, coronary artery calcium (CAC) scoring provides an additional tool for risk refinement. A CAC score of zero is associated with a low short-term risk and may support deferral of pharmacologic therapy in appropriately selected individuals without higher risk conditions, whereas higher CAC values, particularly ≥ 100 Agatston units or ≥ 75th percentile for age and sex, reclassify patients toward higher risk and favor initiation or intensification of lipid-lowering therapy (30). Within a precision medicine framework, CAC scoring enables a more individualized assessment of subclinical atherosclerosis, improving risk discrimination beyond clinical variables alone.

## 12 Primary prevention algorithm

In primary prevention, the intensity of lipid-lowering therapy should be directly proportional to the patient’s baseline cardiovascular risk, making comprehensive cardiovascular risk assessment essential for guiding treatment decisions. For patients classified as very high-risk, high-intensity statin therapy should be initiated as the cornerstone of management. LDL-C levels should be re-assessed 4 to 12 weeks after statin initiation or dose adjustment (42). If the patient on maximally tolerated statin therapy has not achieved the recommended goal of LDL-C below 55 mg/dL, the addition of ezetimibe is recommended as the preferred first-line non-statin therapy (43, 44). This recommendation is based on ezetimibe’s oral administration, established long-term safety profile, and cost-effectiveness relative to other non-statin options (45). Additionally, the Guidelines for the Management of Dyslipidaemia in Patients with Diabetes Mellitus have indicated that, in addition to reducing LDL-C levels, ezetimibe is capable of increasing HDL cholesterol levels by approximately 3–4% (46). For very high-risk primary prevention patients who have persistent LDL-C of 70 mg/dL or higher despite maximally tolerated statin therapy combined with ezetimibe, escalation to a more potent agent should be considered. In this context, either a PCSK9 inhibitor or bempedoic acid represents a viable option to achieve further substantial LDL-C reduction (21, 47). The choice between these agents should be guided by the required magnitude of LDL-C lowering, the patient’s tolerance for an injectable medication, and consideration of inflammatory risk, as bempedoic acid uniquely reduces high-sensitivity C-reactive protein in addition to LDL-C (35). For very high-risk primary prevention patients with confirmed statin intolerance, defined as the inability to tolerate statin therapy due to unacceptable adverse effects particularly muscle symptoms (48), a different therapeutic pathway is required. In this population, initiating therapy with either a PCSK9 inhibitor or ezetimibe represents an effective strategy. Specifically, among statin-intolerant patients with very high cardiovascular risk, adding a PCSK9 inhibitor compared with no lipid-lowering therapy is associated with an absolute reduction of 16 fewer myocardial infarctions and 21 fewer strokes per 1000 patients treated over 5 years, while ezetimibe provides reductions of 11 fewer myocardial infarctions and 14 fewer strokes per 1000 patients over the same period (49).

## 13 Secondary prevention algorithm

Patients with established ASCVD represent the highest priority for intensive lipid-lowering therapy, as they derive the greatest absolute benefit from risk reduction. The paradigm for secondary prevention has evolved toward more aggressive, earlier combination therapy to achieve the stringent LDL-C goal of less than 55 mg/dL as rapidly as possible (30). Consistent with this approach, adding ezetimibe to statin therapy in this population, including those post-acute coronary syndrome, significantly reduces the risk of major adverse cardiovascular events (50). The IMPROVE-IT trial demonstrated that adding ezetimibe to statin therapy after acute coronary syndrome reduced cardiovascular events by 6% over a median follow-up of 6 years (HR 0.936, 95% CI 0.89–0.99) (51). If LDL-C remains ≥ 55 mg/dL despite high-intensity statin plus ezetimibe, a PCSK9 inhibitor should be added (52). The evidence supporting this approach is robust, with the FOURIER trial showing that evolocumab added to statin with or without ezetimibe reduced MACE by 15% over 2.2 years in stable ASCVD (21), and the ODYSSEY OUTCOMES trial showing that alirocumab added to statin after acute coronary syndrome reduced MACE by 15% and demonstrated a 15% reduction in all-cause mortality (26). In high cardiovascular risk patients with LDL-C above 70 mg/dL despite treatment with statin plus ezetimibe, the addition of bempedoic acid provides greater LDL-C lowering than doubling the statin dose and increases the proportion of patients achieving LDL-C goal (53). In the CLEAR Outcomes trial, bempedoic acid reduced MACE by 13% in statin-intolerant patients with ASCVD or high-risk primary prevention (37). Inclisiran, administered as a 284 mg subcutaneous injection at baseline, again at 3 months, and subsequently every 6 months thereafter, provides potent and sustained LDL-C reduction of approximately 50%. This unique dosing schedule, requiring only twice-yearly administration by a healthcare professional, may be particularly advantageous for patients who struggle with adherence to daily oral medications or self-injectable therapies (28). The efficacy and safety of inclisiran have been demonstrated by a pooled patient-level analysis of the ORION-9, 10, and 11 trials reported a placebo-corrected LDL-C reduction of 51% and a favorable, though exploratory, reduction in major adverse cardiovascular events OR (95% CI): 0.74 (0.58–0.94)] (54).

## 14 Precision modifiers for treatment selection

Selection among non-statin therapies should not rely exclusively on LDL-C levels. A precision medicine approach should also consider treatment adherence, residual inflammatory risk, route of administration, expected magnitude of LDL-C reduction, post-acute coronary syndrome status, genetic background, previous intolerance to lipid-lowering therapy, and local drug availability. In general, ezetimibe is the preferred first add-on therapy because of its oral administration, favorable safety profile, and cost-effectiveness. PCSK9-targeted therapies are preferred when a larger LDL-C reduction is required, particularly in very high-risk patients or those with familial hypercholesterolemia. Inclisiran may be especially useful in patients with adherence difficulties because of its twice-yearly dosing schedule. Bempedoic acid is relevant in patients with confirmed statin intolerance or those unable to achieve LDL-C targets despite optimized statin therapy, as well as in those with residual inflammatory risk, given its effects on both LDL-C and hsCRP.

## 15 Implementation considerations in Latin America

A recent systematic review and meta-analysis on the global prevalence of dyslipidemia reported that the prevalence of hypercholesterolemia was 68.19% in Ecuador, 49.82% in Chile, 49.75% in Peru, 43.57% in Brazil, 34.10% in Colombia, 29.03% in Argentina, and 22.09% in Mexico (5). Regarding hypertriglyceridemia, the highest prevalence was reported in Colombia (53.07%), followed by Ecuador (34.56%), Argentina (31.57%), Chile (31.24%), Peru (29.73%), and Brazil (7.79%) (5).

In Latin America, implementation of precision dyslipidemia management must account for marked heterogeneity in healthcare systems, drug availability, reimbursement policies, and patient out-of-pocket costs. For this reason, treatment algorithms should be interpreted within local clinical and regulatory contexts. In many settings, ezetimibe may remain the most accessible first-line non-statin option, whereas PCSK9 inhibitors and inclisiran may be restricted by cost or availability. In Brazil, it is estimated that nearly 90% of individuals with familial hypercholesterolemia receive high-intensity lipid-lowering therapy; however, the use of PCSK9 inhibitors remains extremely low (2%). In Mexico, only 7.5% of patients are treated with PCSK9 inhibitors (55). Similarly, a study by Ramírez-Peña et al. reported that only 7,431 prescriptions for these agents were issued in Colombia over a 3-year period, despite a population of nearly 50 million inhabitants (56). In Latin America, approximately 78% of all medications are paid for out of pocke (57). These findings highlight the substantial underutilization of PCSK9 inhibitors across Latin America. Thus, precision medicine in the region should integrate not only biological and clinical risk factors, but also treatment feasibility, adherence potential, and health-system realities.

## 16 Limitations

This study has several limitations. First, as an expert positioning rather than a formal guideline developed using structured evidence-grading systems, our recommendations may be influenced by expert consensus and lack a standardized hierarchy of evidence. Second, we did not include pharmacoeconomic analyses, which are particularly relevant in Latin America, where access, affordability, and health system constraints strongly influence treatment decisions. Third, much of the evidence informing our recommendations is derived from randomized clinical trials conducted outside the region, which may limit generalizability to real-world settings across diverse Latin American populations. Fourth, our focus on LDL-C as the primary therapeutic target does not fully capture the multifactorial nature of cardiovascular risk, including other lipid fractions and non-lipid determinants. Additionally, the absence of direct head-to-head comparisons between non-statin therapies limits the strength of comparative treatment recommendations. Finally, the implementation of precision medicine approaches may be constrained by variability in healthcare infrastructure, availability of advanced therapies, and access to diagnostic tools across countries in the region.

## 17 Conclusion

Non-statin therapies enable precision dyslipidemia management in the era of strict targets, with ezetimibe as first-line add-on, PCSK9 inhibitors for high-risk persistence, and bempedoic acid for statin intolerance. Algorithms prioritize risk stratification, inflammation, and accessibility in Latin America. Future research should evaluate long-term mortality and cost-effectiveness.
